# Worry experienced during the 2015 Middle East Respiratory Syndrome (MERS) pandemic in Korea

**DOI:** 10.1371/journal.pone.0173234

**Published:** 2017-03-08

**Authors:** Jun-Soo Ro, Jin-Seok Lee, Sung-Chan Kang, Hye-Min Jung

**Affiliations:** 1 Department of Infectious Disease, Research Center of Infectious and Environmental Disease, Armed forces medical research institute, Daejeon, South Korea; 2 Department of Health Policy and Management, College of Medicine, Seoul National University, Seoul, South Korea; 3 Institute of Health Policy and Management, Medical Research Center, Seoul National University, Seoul, South Korea; Shanxi University, CHINA

## Abstract

**Background:**

Korea failed in its risk communication during the early stage of the Middle East Respiratory Syndrome (MERS) outbreak; consequently, it faced difficulties in managing MERS, while disease-related worry increased. Disease-related worry can help disease prevention and management, but can also have a detrimental effect. This study measured the overall level of disease-related worry during the MERS outbreak period in Korea and the influencing factors and levels of disease-related worry during key outbreak periods.

**Methods:**

The cross-sectional survey included 1,000 adults who resided in Korea. An ordinal logistic regression was performed for the overall level of MERS-related worry, and influencing factors of worry were analyzed. A reliability test was performed on the levels of MERS-related worry during key outbreak periods.

**Results:**

The overall level of MERS-related worry was 2.44. Multivariate analysis revealed that women and respondents w very poor subjective health status had higher levels of worry. Respondents with very high stress in daily life had higher levels of worry than those who reported having little stress. The reliability test results on MERS-related worry scores during key outbreak periods showed consistent scores during each period.

**Conclusion:**

Level of worry increased in cases having higher perceived susceptibility and greater trust in informal information, while initial stage of outbreak was closely associated with that at later stages. These findings suggest the importance of managing the level of worry by providing timely and accurate disease-related information during the initial stage of disease outbreak.

## Introduction

Korea recently experienced an outbreak of Middle East Respiratory Syndrome (MERS). According to the Korea Center for Disease Control and Prevention (KCDC), 186 people were definitively diagnosed with MERS between May 20 and November 30, 2015; of those, 38 died from the disease. The people diagnosed with MERS consisted of 82 hospital patients, 65 family member or visitors, and 39 hospital workers. Moreover, 16,752 individuals were quarantined for infection management [1]. Simulating the disease spread process during the period of MERS by using a mathematical model, the result was 4.42 R0 value that indicating infectivity [2]. According to the criteria set by the World Health Organization (WHO), the MERS pandemic ended by late December; however, the Korean government actually declared late July as the end of the pandemic.

The Korea-WHO MERS Joint Mission determined that the MERS outbreak in Korea was due to initial response failure by the Korean government. The cause of this initial response failure was attributed a lack of transparent disclosure and timely information [3].

According to the Crisis and Emergency Risk Communication (CERC) guidelines from the US Center for Disease Control and Prevention (CDC), when a national crisis, such as infectious epidemic, occurs, the most important action is timely dissemination spread of information and communication. However, the Korean government was unable to disclose information immediately after the MERS outbreak, which resulted in outbreak spread and increased levels of worry about MERS among its citizens [4].

Disease-related worry plays an important role in disease management, especially with contagious disease management. Disease-related worry is an emotional response to a disease and it is known to have a positive effect on disease management by providing individuals with motivation to participate in health promotion activities [5–7]. However, at times, disease-related worry can result in stigmatization, which can have a negative effect on disease management.

Due to the government’s failure to share timely information in the early stage of the MERS outbreak in Korea, the outbreak increased worries among Korean citizens and presented difficulties in disease management. Therefore, in order to effectively manage future disease outbreaks, it is important to understand the levels of disease-related worry and its influencing factors. Moreover, assessment of the levels of disease-related worry by key outbreak periods may also inform effective intervention points.

**Fig 1:**
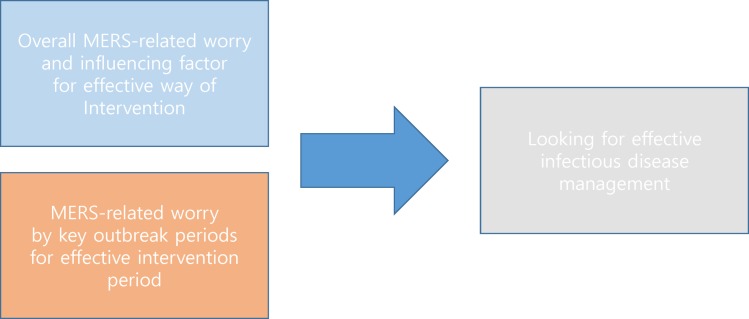
Structure of the study.

However, virtually no studies to date in Korea have examined disease-related worry, particularly contagious disease-related worry, during disease outbreak and its influencing factors. To our knowledge, no study has investigated disease-related worry according to specific outbreak periods. Accordingly, the objective of the present study was to identify the overall level of MERS-related worry during the MERS outbreak period as well as the levels of disease-related worry during key MERS outbreak periods. Structure of the study is shown in Fig 1.

## Materials and methods

### Participants

The participants in this study consisted of 1,000 adults more than 18 years old who resided in Korea. The sample was selected by proportional stratified sampling between panels of professional research agency (Research & Research Cooperation). For the representativeness, the sample proportion of was determined by sexual, regional, age population ratio in Korea. Anyone who submitted his or her E-mail address and reply 2–3 simple questions via website of our research agency could be a panel. The survey was conducted in an online format from August 24^th^, 2015 to September 11^th^, 2016.

This study received a review exemption from the institutional review board (IRB) of Seoul National University Hospital (IRB No.1508-064-694).

### Survey instrument

So the dependent variables in the study were the overall level of worry during the MERS outbreak and the level of worry during specific outbreak periods. The overall level of worry during the MERS outbreak was surveyed with a five-point scale, using the question (S2 and S3 Files), “Have you ever worried about being infected with MERS during the MERS outbreak period?” The level of MERS-related worry according to specific outbreak periods was also surveyed with a five-point scale, using the question (S2 and S3 Files), “Did you worry about being infected with MERS when the first patient with MERS had a definitive diagnosis/when the first patient with MERS died/when the number of patients continued to rise/when MERS outbreak ended?”. The MERS-related worry question was based on a question used in a precedent study [8].

Demographic variables for identification of participant characteristics included age, sex, education level, average income per month, type of medical insurance and occupation type. In addition, to assess the physical and mental status of the participants, self-rated health status, chronic disease status (hypertension, diabetes) and daily life stress level were surveyed (S2 and S3 Files). The Survey conducted by classified age groups: ‘19–29’, ‘thirties’, ‘forties’, ‘fifties’, ‘over sixties’. In this analysis, Age groups are categorized as ‘under 29’, ‘over thirty under forty’ and ‘over fifty’ following by precedent study [9]. The Survey answer types of a education level were classified in ‘below middle school graduation’, ‘high school graduation’, ‘College student or graduation’ and ‘graduate school student or graduation’. In this analysis, Education levels are categorized as ‘below high school graduation’, ‘College student or graduation’, and ‘graduate school student or graduation’. The classification needed is necessary in this study because the college entrance rate is high (approximately 70%) in Korea. The Survey answer types of an occupation were categorized in ‘Agriculture/Fishing/Forestry/Animal Husbandry’, ‘Self-employment’, ‘Sales/Service’, ‘Technical engineer/Skilled labor’, ‘Simple labor(Production employee, Sweeper, Janitor etc.)’, ‘Office job(Office worker, Public officer etc.)’, ‘Manager’, ‘Specialized job(Lawyer, Doctor, Architect, Professor etc.)’, ‘Housewife’, ‘Student’ and ‘unemployed’. In this analysis, the three of occupations: ‘office job’, ‘Manager’ and ‘specialized job’ are classified as a white collar worker. ‘Housewife’, ‘student’ and ‘unemployed’ are classified as an ‘unemployed’. The other occupations are classified as a blue collar worker. The Survey answer types of medical insurance status were categorized in ‘Regional medical insurance’, ‘Workplace medical insurance’ and ‘Medical Aid’. In this analysis, medical insurance statuses are categorized in ‘Medical insurance’ or ‘Medical Aid’. The Survey answer types of income per a month were categorized in ‘Below 1,000$’, ‘1,000$-2,000$’, ‘2,000$-3,000$’, ‘3,000$-4,000$’, ‘4,000$-5,000$’, ‘5,000$-6,000$’, ‘Over 6,000$’. In this analysis, incomes are categorized in ‘Under 2,000$’, ‘2,000$-4,000$’, ‘4,000$-6,000$’, ‘Over 6,000$’ following by income quartile in Korea. The five-point scale survey conducted on Self-rated health status followed by quartile of income status in Korea. In this survey, if respondents have hypertension or diabetes, they categorized in ‘Chronic disease present’. Stress level in daily life was surveyed with a five-point scale.

### Statistical analysis

A univariate analysis using t-test and one-way analysis of variance (AVOVA) was performed to examine the relationships between the overall level of MERS-related worry and each independent variable. Moreover, to control for confounding variables, a multivariate analysis was performed through an ordinal logistic regression. The reliability of the level of MERS-related worry by outbreak periods was analyzed via Fleiss Kappa and intra-class correlation (ICC). IBM SPSS Statistics for Windows, version 20.0 was used for all analyses.

## Results

The results of univariate analysis between the general characteristics of the participants and the overall level of MERS-related worry are shown in Table 1. The mean MERS-related worry score, scored on a five-point scale, was 2.44 points. In the univariate analysis, sex, self-rated health status, and daily life stress level showed statistically significant results. Men showed a lower overall level of MERS-related worry than women did. The group of participants that reported ‘very good’ health status showed a lower overall level of MERS-related worry than the groups that responded ‘bad’, ‘normal’, and ‘good’. Moreover, the group that responded ‘little’ to daily life stress had a lower level of MERS-related worry than group that responded ‘very much’.

**Table 1:** General characteristics of the participants and univariate analysis.

Characteristics	N(%)	MERS^a^-related worry (five-point scale)	P-value
Age (years)			
• <29 • 30–49 • 50<	• 231(23.1%) • 507(50.7%) • 262(26.2%)	• 2.45 • 2.47 • 2.37	0.322
Sex			
• Male • Female	• 520(52.0%) • 480(48.0%)	• 2.36 • 2.53	0.003
Self-rated health status			
• Very bad • Bad • Normal • Good • Very good	• 12(1.2%) • 126(12.6%) • 372(37.2%) • 429(42.9%) • 61(6.1%)	• 1.92 • 2.48 • 2.52 • 2.42 • 2.10	0.002
Medical insurance			
• Medical aid • Health insurance	• 76(7.6%) • 924(92.4%)	• 2.32 • 2.45	0.280
Education			
• Below high school graduation • College graduation • Graduate school	• 177(17.7%) • 733(73.3%) • 90(9.0%)	• 2.32 • 2.48 • 2.38	0.088
Income (per month)			
• <$2,000 • $2,000-$4,000 • $4,000-$6,000 • $6,000<	• 132(13.2%) • 385(38.5%) • 332(33.2%) • 151(15.1%)	• 2.32 • 2.52 • 2.44 • 2.35	0.072
Stress in daily life			
• Little • Not much • Much • Very much	• 21(2.1%) • 348(34.8%) • 515(51.5%) • 116(11.6%)	• 1.95 • 2.38 • 2.51 • 2.39	0.007
Chronic disease (HTN^b^, DM^c^)			
• Not present • Present	• 828(82.8%) • 172(17.2%)	• 2.46 • 2.37	0.228
Job			
• Blue-collar • White-collar • Unemployed	• 220(22.0%) • 449(44.9%) • 331(33.1%)	• 2.40 • 2.42 • 2.49	0.388

^a^MERS, Middle East Respiratory Syndrome

^b^HTN, Hypertension

^c^DM, Diabetes Mellitus

Ordinal logistic regression analysis results also revealed statistically significant associations with overall level of MERS-related worry with respect to sex, self-rated health status, and daily life stress, just as in the univariate analysis. Men showed lower odds ratios (ORs) than women. The group that responded as having ‘very good’ self-rated health status showed lower ORs than all other groups, except for the group that responded ‘very bad’. Moreover, the group that responded as having ‘little’ daily life stress showed lower ORs than the group that responded as having ‘very much’ daily life stress (Table 2). These results satisfied the parallel assumptions of ordinal logistic regression.

**Table 2:** Ordinal regression analysis of degree of MERS^a^-related worry and independent predictors.

Characteristics	B(SE^e^)	ORs^d^	P-value
Age (years)			
• <29 • 30–49 • 50<	• 0.075(0.187) • 0.120(0.148) • 0.000	• 1.078 • 1.127 • 1.000	• 0.688 • 0.419 • -
Sex			
• Male • Female	• -0.319(0.130) • 0.000	• 0.727 • 1.000	• 0.014 • -
Self-rated health status			
• Very Bad • Bad • Normal • Good • Very Good	• -0.195(0.622) • 0.825(0.307) • 0.901(0.271) • 0.700(0.264) • 0.000	• 0.823 • 2.282 • 2.462 • 2.014 • 1.000	• 0.754 • 0.007 • 0.001 • 0.008 • -
Medical insurance			
• Medical aid • Health insurance	• -0.287(0.237) • 0.000	• 0.751 • 1.000	• 0.226 • -
Education			
• Below high school graduation • College graduation • Graduate school	• -0.140(0.258) • 0.208(0.212) • 0.000	• 0.869 • 1.231 • 1.000	• 0.588 • 0.325 • -
Income (per month)			
• <$2,000 • $2,000-$4,000 • $4,000-$6,000 • $6,000<	• -0.080(0.237) • 0.298(0.187) • 0.142(0.185) • 0.000	• 0.923 • 1.347 • 1.153 • 1.000	• 0.736 • 0.111 • 0.442 • -
Stress in daily life			
• Little • Not much • Much • Very much	• -1.106(0.463) • 0.011(0.209) • 0.263(0.197) • 0.000	• 0.331 • 1.011 • 1.301 • 1.000	• 0.017 • 0.957 • 0.182 • -
Chronic disease (HTN^b^, DM^c^)			
• Present • Not present	• 0.167(0.167) • 0.000	• 1.182 • 1.000	• 0.319 • -
Job			
• Blue-collar • White-collar • Unemployed	• -0.154(0.174) • -0.127(0.154) • 0.000	• 0.857 • 0.881 • 1.000	• 0.379 • 0.408 • -

^a^MERS, Middle East Respiratory Syndrome

^b^HTN, Hypertension

^c^DM, Diabetes Mellitus

^d^ORs, Odds Ratios

^e^SE, Standard Error

The levels of MERS-related worry by MERS outbreak periods showed scores of 2.32, 2.45, 2.73, and 2.24 when the first patient with MERS had a definitive diagnosis (index case), the first patient with MERS died (first death), the number of patients continued to rise (increasing patients), and the MERS outbreak ended (end of outbreak), respectively (Table 3).

**Table 3:** Worry during key outbreak periods.

Key periods	Levels of MERS-related worry					
	1	2	3	4	5	Average
	Not worried				Very worried	
Index case	197(19.7%)	395(39.5%)	316(31.6%)	75(7.5%)	17(1.7%)	2.32
First death	165(16.5%)	379(37.9%)	326(32.6%)	104(10.4%)	26(2.6%)	2.45
Increasing patients	114(11.4%)	290(29.0%)	393(39.3%)	159(15.9%)	44(4.4%)	2.73
End of outbreak	246(24.6%)	391(39.1%)	262(26.2%)	78(7.8%)	23(2.3%)	2.24

The scores from the levels of MERS-related worry by MERS outbreak periods were compared via a reliability test. Fleiss Kappa and intra-class correlation test results showed that worry scores from each period were statistically significantly consistent (Table 4).

**Table 4:** Reliability test of worry during key outbreak periods.

	Coefficient	P-value
Fleiss Kappa	0.430	<0.001
Intra class correlation	0.689	<0.001

## Discussion

The objective of the present study was to investigate the overall level of MERS-related worry among the Korean population during the MERS outbreak, its influencing factors, and levels of worry during key outbreak periods, in order to assist management of future disease outbreaks.

The mean overall MERS-related worry score was 2.44 points. According to a 2006 study by Chapman et al., when American college employees were surveyed during 2001–2002 regarding their worry about influenza, the level of worry ranged from 1.93 to 2.24 points, depending on whether the person had been vaccinated for influenza [10]. In comparison, the level of MERS-related worry in Korea was higher than that of level of worry associated with infection by usual respiratory diseases.

Until now, no precedent studies have reported on MERS-related worry. However, many precedent studies have investigated the level of worry during influenza A (H1N1) pandemics. As these studies used different instruments from the ones used in this study, it couldn’t be compared directly. However, the overall trend showed higher levels of worry than the MERS-related worry found in the present this study. In a study conducted in the Netherlands, a five-point scale was used to investigate the level of worry, just as that used in the present study [9]. In that study, 36% of the respondents reported feeling worried to very worried in 36%, a rate higher than the 9.2% of worried to very worried responses during the first period (index case observed) in the present study. In a study conducted in Saudi Arabia, the level of concern was investigated at three intervals, and a relatively high response ‘extremely concerned’ (54.3%) was observed among respondents [11]. A study conducted in Malaysia and Europe investigated the levels of concern in four intervals and found that 26% and 25% felt very concerned and somewhat concerned, respectively [12].

Ordinal logistic regression was used to analyze the influencing factors of the overall level of MERS-related worry, revealing statistically significant results for sex, self-rated health status, and daily life stress. With respect to sex, men showed lower ORs than women, indicating higher level of MERS-related worry among women. This finding was consistent with the results of a precedent study [13]. The level of worry was likely higher in women was because they have higher perceived susceptibility. Precedent studies have perceived susceptibility to be higher in women than in men, and higher perceived susceptibility results in increased disease-related worry [14, 15].

The group that reported their health status to be ‘very good’ had lower ORs than all other groups, a finding that can be interpreted two ways. First, the group that responded as having very good self-rated health status had confidence in their physical health, thereby having a lower perceived susceptibility than the other groups. The second reason may be attributed to differences in the level of trust in informal information. According to a precedent study, having greater trust in informal information correlates with increased levels of disease-related worry [15]. The group that reported having very good self-rated health status had greater interest in health than the other groups; as a result, they may have increased interest in the situation involving the MERS outbreak and acquiring more information on the disease and methods for its prevention. Because of this, they had less trust in informal information, which can result in lower levels of MERS-related worry.

The group that reported having very high daily life stress showed higher ORs than the group that reported having little stress. This suggests that the group with very high daily life stress was mentally vulnerable and more sensitive to the MERS outbreak.

Investigation of level of worry by key outbreak periods demonstrated that the level of worry increased from the point of the index case, to the first death, and to the period when the disease was spreading. The level of worry then decreased at the point of outbreak ending. The reliability test results showed statistically significantly consistent for scores from all four periods. This finding can be interpreted to mean that the level of worry in the initial stage was closely associated with the level of worry in the stages that followed.

Increase in the level of worry over time contradicts findings in other studies. According to a study from the Netherlands conducted during an influenza A (H1N1) pandemic, the level of worry was highest in the initial stage, after which the level decreased and was maintained at a consistently low level. A study from Hong Kong also indicated that the level of perceived worry was high initially, but continued to decrease thereafter. The reason for these somewhat conflicting results in comparison to precedent studies is because the influenza A (H1N1) outbreak was touted early on by the government as a very serious disease, which resulted in high levels of worry. However, as people gradually realized that the disease was not much different than any previous influenza outbreaks, the level of worry decreased accordingly [10, 16]. In contrast, in the early stage of MERS outbreak in Korea, it was announced that the disease was not any more serious than other influenzas; however, the increasing number of infected patients and mortalities is believed to have resulted in increased levels of worry. These findings can be used as evidence that effectively reducing the level of worry in the population during the early stage would result in a persistently low level of worry.

Precedent studies on emerging respiratory infectious disease also emphasize the importance of formal outbreak prevention information. A 2010 study by Cowling et al. attributed the lack of risk communication as the reason why the initial perceived susceptibility in the population did not change even when the 2009 influenza A (H1N1) outbreak in Hong Kong continued to spread [16], while a 2009 study by Raude et al [13]. indicated that not providing sufficient information during the pandemic of influenza A (H1N1) had a negative effect on the prevention and spread of influenza. In particular, a 2009 study on the swine flu, conducted by Rubin et al., reported that providing people with practical information to increase their knowledge of infection prevention and outbreak is important, and that emphasizing recommended preventative measures and possible outbreak duration can be helpful in managing the disease outbreak. These study results are in agreement with the findings of the present study [17].

The public health implications of this study are as follows. It is effective to control outbreaks by lowering the perceived susceptibility and successfully controlling the spread of informal information in the early stage of the epidemic. Recent research has shown that the changes of spatial diffusion of disease predict an epidemic, so it can be using early warning indicators. Therefore, continual checking the spatial spread of disease at the every stage is helpful to disease-related worry intervention [18]. According to the Suggestion from the precedent study that analyzing the MERS epidemic in Korea through mathematical model, the interventions; limiting the theatrical performances such as rallies and fairs, publishing information on diseases at appropriate times and distributing them to the local community could also be helpful to manage of disease. Through these interventions, it is possible to lowering down the perceived susceptibility rate and preventing the spread of informal information [2].

The present study had several limitations. First, the present study was a cross-sectional study; as such, it may contain recall bias, which can especially influence the level of MERS-related worry during key outbreak periods. Because the worry scores for the four different periods were surveyed simultaneously, approximately three months had elapsed from the time of the index case to when the survey was conducted; therefore, the levels of worry during the corresponding periods may not have been recalled accurately, thereby resulting in inaccurate responses. Future studies should conduct prospective surveys throughout the duration of infection outbreaks in order to more accurately assess the levels of disease-related worry during key outbreak periods. Second, emotional responses to epidemics can be assessed using various questions. Besides the experienced worry assessed in the present study, various other questions, including anxiety level, may offer a deeper understanding of the emotional responses to epidemics.

## Conclusions

This study analyzed factors associated with level of worry during the MERS outbreak period. The factors that decreased the level of MERS-related worry were being men and having very good self-rated health status, whereas having very high daily life stress increased the level of worry. These findings were to suggest that the level of worry increased among those with higher perceived susceptibility and with greater trust in informal information. Moreover, with respect to the levels of worry according to MERS outbreak periods, the level of worry in the initial stage was closely associated with the levels during the stages that followed. This observation underscores the importance of managing the level of worry in the early stage of outbreaks through proper intervention. Therefore, for proper disease management, it is necessary to make efforts to reduce the level of perceived susceptibility by disseminating timely and accurate information on disease prevention measures and current status through formal routes in the early stage of the outbreak, in addition to reducing the level of worry in the early stage by blocking distribution of informal information. For this, it is helpful to outbreak control by publishing disease information at the appropriate time and distributing it to the community, and to educate each group about disease prevention in a personalized manner.

## Supporting information

S1 File Minimal dataset for the analyses. (XLS)

S2 File Questionnaire (in English). (PDF)

S3 File Questionnaire (in Korean). (PDF)
