# Supplementary material for: Worry experienced during the 2015 Middle East Respiratory Syndrome (MERS) pandemic in Korea
Source: PLoS One. 2017 Mar 8;12(3):e0173234. doi: 10.1371/journal.pone.0173234 (PMC5342218; doi:10.1371/journal.pone.0173234)
Supplement: S2 File — (PDF) [file pone.0173234.s002.pdf]

Research & Research

Survey about health care

Hello? This is the survey for perception about health care, society, medicine between the populations. We appreciate for your valuable time. We will use every answer you submitted for nothing but the research under the statistical law section 33 and 34.

2015. 08

Research & research (R&R) (Corp.)

SQ1. What is your sex?

1. Male 2. Female

SQ2. What is your age?

1. 19~29 2. 30s 3. 40s 4. 50s 5. over 60

● **Life style and behavior**

Q01. How do you feel about your health status?

① Very good ② Good ③ So So

④ Bad ⑤ Very bad

Q02. How much do you feel stress in your daily life?

① Very much ② Quite ③ So So

③ Not much ④ Few

Q03. Have you ever diagnosed as following disease from medical doctor? (Multiple answers allowed)

① Hypertension	② Hyperlipidemia	③ Stroke	④ Angina·MI
⑤ Osteoarthritis/Rheumatoid arthritis	⑥Pulmonary tuberculosis	⑦ Asthma	⑧ Diabetes
⑨ Hyper/Hypothyroidism	⑩ Cancer	⑪ Mental disorder (Depression etc.)	⑫ Renal dysfunction
⑬ Liver disease	⑭ Etc.	⑮ No diagnosis	

● **MERS related worry**

Q4. Have you ever worried about being infected with MERS during the MERS outbreak period?

① Never thought about it ② Thought about it but it wasn't worries

③ Worried me a bit ④ Worried me a lot

⑤ Worried about it all the time

Q5. Questions about MERS worry during outbreak periods

1.	2.	Never thought about it	Thought about it but it wasn't worries	Worried me a bit	Worried me a lot	Worried about it all the time
Q5_1	Did you worry about being infected with MERS when the first patient with MERS had a definitive diagnosis	①	②	③	④	⑤
Q5_2	Did you worry about being infected with MERS when the first patient with MERS died	①	②	③	④	⑤
Q5_3	Did you worry about being infected with MERS when the number of patients continued to rise	①	②	③	④	⑤
Q5_4	Did you worry about being infected with MERS when MERS outbreak ended	①	②	③	④	⑤

DQ1. What kind of medical insurance do you have?

① Regional medical insurance

② Workplace medical insurance

③ Medical Aid (Type I, II)

DQ2. What is your education level?

① Below middle school graduation

② High school graduation

③ College student/graduation

④ Graduate school student/graduation

⑤ No answer

DQ3. What is your occupation?

① Agriculture/Fishing/Forestry/Animal Husbandry

② Self-employment (Merchandise, Private taxi driver etc.)

③ Sales/Service (Shop assistant, Salesman, Door-to-door salesman, Hairdresser etc.)

④ Technical engineer/Skilled labor (Bus/Taxi driver, Laundry, Carpenter, Engineer 등)

⑤ Simple labor (Production employee, Sweeper, Janitor etc.)

⑥ Office job (Office worker, Public officer etc.)

⑦ Manager

⑧ Specialized job (Lawyer, Doctor, Architect, Professor etc.)

⑨ Housewife

⑩ Student

⑪ Unemployed

⑫ Etc. (   )

⑬ No answer

DQ4. How much is your family income per a month?

① Under 1,000$ ② 1,000$~2,000$ ③ 2,000$-3,000$

④ 3,000$-4,000$ ⑤ 4,000$~5,000$ ⑥ 5,000$~6,000$

⑦ Over 6,000$

⑧ No answer

♣ Thank you ♣
