# Supplementary material for: Worry experienced during the 2015 Middle East Respiratory Syndrome (MERS) pandemic in Korea
Source: PLoS One. 2017 Mar 8;12(3):e0173234. doi: 10.1371/journal.pone.0173234 (PMC5342218; doi:10.1371/journal.pone.0173234)
Supplement: S3 File — (PDF) [file pone.0173234.s003.pdf]

의료 관련 설문조사

안녕하세요? 본 조사는 서울대학교 의과대학의 의뢰로 일반 국민들의 건강 분야 및 사회, 의학 분야에 대한 인식을 파악하기 위한 연구 목적이니 바쁘시더라도 잠시만 시간을 내어 주시면 고맙겠습니다. 응답하신 모든 내용은 통계법 33조 및 34조에 따라 연구 목적 외에는 절대로 다른 용도로 사용되지 않을 것입니다.

2015. 08

(주)리서치앤리서치(R&R)

SQ1. 성별

1. 남자   2. 여자

SQ2. 선생님의 연령은 올해 만으로 어떻게 되십니까? 만 (   ) 세

1. 만19~29세   2. 30대   3. 40대   4. 50대   5. 60세 이상

● **지금부터 생활 습관 및 행태와 관련하여 여쭙겠습니다.**

Q01. 평소 귀하의 건강 상태는 어떻다고 생각하십니까?

① 매우 건강하다   ② 건강한 편이다   ③ 보통이다

④ 건강하지 않은 편이다   ⑤ 전혀 건강하지 않다

Q02. 귀하께서는 평소 일상생활 중에 스트레스를 어느 정도 느끼고 있습니까?

① 매우 많이 느낀다 ② 많이 느끼는 편이다

③ 별로 느끼지 않는 편이다 ④ 거의 느끼지 않는다

Q03. 귀하께서는 다음 질환을 **의사로부터 진단**받은 적이 있습니까? (복수 응답)

①고혈압	②고지혈증	③뇌졸중(중풍)	④협심증·심근경색증
⑤골관절염·류마티스성관절염	⑥폐결핵	⑦천식	⑧당뇨병
⑨갑상선 장애(항진증, 저하증 등)	⑩암	⑪우울증 등 정신질환	⑫신부전
⑬간경변증	⑭기타 질환	⑮진단받은 적 없음	

● **전염 질환(메르스) 관련 인식**

Q4. 메르스 유행기간 동안에 **본인이 메르스에 걸릴 것이라고 어느 정도 걱정하였습니까?**

① 생각해 본 적도 없다 ② 생각은 해 봤지만 걱정하지 않았다

③ 약간 걱정했다 ④ 많이 걱정했다

⑤ 항상 걱정했다

Q5. 다음은 메르스 유행 시기별 질문입니다.

1.	2.	3. 전혀 걱정하지 않았다	4. 별로 걱정하지 않았다	5. 약간 6. 걱정 했다	7. 많이 8. 걱정 했다	9. 항상 10. 걱정 했다
Q5_1	**메르스 첫 환자가 발생했을 때,** 본인이 메르스에 걸릴 것이라고 어느 정도나 걱정하였습니까?	①	②	③	④	⑤
Q5_2	**메르스 첫 사망자가 발생했을 때,** 본인이 메르스에 걸릴 것이라고 어느 정도나 걱정하였습니까?	①	②	③	④	⑤
Q5_3	**메르스 환자 발생이 급격히 늘었을 때,** 본인이 메르스에 걸릴 것이라고 어느 정도나 걱정하였습니까?	①	②	③	④	⑤
Q5_4	**정부가 메르스 사태 종결을 발표했을 때,** 본인이 메르스에 걸릴 것이라고 어느 정도나 걱정하였습니까?	①	②	③	④	⑤

DQ1. 귀하께서는 어떤 종류의 건강 보험에 가입되어 있습니까?

① 지역 건강보험

② 직장 건강보험

③ 의료급여(1, 2종)

DQ2. 실례지만 학교는 어디까지 마치셨습니까?

① 중졸이하

② 고졸

③ 대학교 재학/졸업

④ 대학원 재학/졸업 이상

⑤ (불러주지 말 것) 없음/모름/무응답

DQ3. 선생님의 직업은 무엇입니까?

① 농업/임업/어업/축산업

② 자영업 (상업, 소규모 장사, 개인택시운전사 등)

③ 판매/서비스직 (상점 점원, 세일즈맨, 방문 판매원, 미용사 등)

④ 기능/숙련공 (운전기사, 세탁, 선반, 목공, 기능공 등)

⑤ 일반작업직 (제조업 생산직, 현장직업, 일용노무직, 청소관리, 경비원 등)

⑥ 사무기술직 (차장이하 사무직, 초중고 교사, 6급이하 공무원)

⑦ 경영/관리직 (5급이상 공무원, 기업체 부장 이상)

⑧ 전문/자유직 (변호사, 의사, 건축사, 교수, 예술가, 종교지도자 등)

⑨ 가정주부 (가사와 육아만 하는 전업주부)

⑩ 학생

⑪ 무직

⑫ 기타(   )

⑬ 모름/무응답

DQ4. 선생님 댁 가족 전체의 보너스를 포함한 월평균 소득은 얼마입니까?

① 100만원 이하   ② 100~200만원 미만   ③ 200~300만원 미만

④ 300~400만원 미만   ⑤ 400~500만원 미만   ⑥ 500~600만원 미만

⑦ 600만원 이상

⑧ 모름/무응답

♣ 감사합니다. ♣
